# Countermeasures for Urban Traffic Congestion in China from the Perspective of System Dynamics

**DOI:** 10.1155/2022/3509902

**Published:** 2022-03-24

**Authors:** Wenjing Zhang

**Affiliations:** School of Economics and Management, Beijing Jiaotong University, Beijing 100044, China

## Abstract

In recent years, urban traffic congestion has seriously affected the healthy development of urbanization in China. And many measures to combat congestion have had little effect . The purpose of this paper is to find out the most reasonable and sustainable measures to control traffic congestion . Based on the theory of system dynamics, this study constructs a model of the formation mechanism of urban traffic congestion in China, and analyzes the thinking error of the traditional strategy of “building roads to eliminate traffic congestion” This study includes the current policy measures to control traffic congestion in the system dynamics model and analyzes the influence of each measure on the formation mechanism of urban traffic congestion. Then, it critiques the unsustainability of rigid policies, such as the vehicle number limit and the “similar road building to control traffic congestion” policies. This study reveals that of the five policies adopted by the government to alleviate traffic congestion, and come to the conclusion: the “sparse block collocation” policy is the most sustainable and fundamental congestion control measure. To achieve efficient traffic congestion control and support the healthy development of urbanization in China in the future, the government should increase the balance of infrastructure investment to improve the slow environment of public transport, adhere to public transport-oriented land development policies, raise the cost of motor vehicle travel, and promote urban traffic.

## 1. Introduction

In recent years, the problem of urban traffic congestion in China has become increasingly serious. Traffic congestion first appeared in megacities such as Beijing, Shanghai, and Guangzhou, and quickly spread to smaller cities. Urban traffic congestion not only delays people's normal travel and damages their physical and mental health [1], but also reduces the efficiency of urban economic life and energy use and intensifies greenhouse gas emissions, which are serious threats to the healthy development of urban areas in China. Therefore, it is urgent to put forward and implement reasonable and sustainable measures to control the congestion. And the purpose of this study is to identify the most reasonable and sustainable treatment measure. Discussions about urban traffic congestion in academia and society have included rail transit, intelligent transportation, citizen quality, development stage, population, bus, and system theories. These theories have been used to determine the causes of urban traffic congestion from their specific fields and personal experiences to provide corresponding countermeasures. For example, Minghua and Zhenghe studied techniques to solve traffic problems in big cities and found that effective countermeasures should be taken to adjust the urban traffic volume from the two principles of reasonably reducing traffic occurrence and improving traffic efficiency as much as possible [2]. Yuanqing et al. studied institutional causes and solutions of traffic problems in China's big cities and examined the role of urban traffic's sustainable development from the perspective of the law of urban traffic development [3]. Xianglin et al. explored coupled mapping following model and traffic congestion control based on an intelligent transportation system [4]. As Reported by the Nanfang Daily, National People's Congress deputies stated that a large number of cars was not the root cause of congestion [5]. Feng et al. studied how to regulate the population scale in megacities, and believed that populations in the main urban areas should be evacuated, infrastructure investment should be increased, and urban industrial structures and economic development speed should be adjusted [6]. Taking Nanjing as an example, Xianteng et al. explored the contradiction between the supply and traffic demand in big cities and development countermeasures and believed that the fundamental root of traffic congestion was the imbalance between the traffic supply and demand [7]. The above studies suggested the causes of urban traffic congestion from different perspectives and proposed the corresponding governance measures.And the findings of the previous research provides reference and inspiration for this study. However, these studies lack critical synthesis and have not provided any detailed analysis of urban traffic congestion from a systematic viewpoint. Therefore, from the perspective of system dynamics (SD), the current study provides a detailed analysis of urban traffic congestion and its governance measures, which is innovative to a certain extent and is of certain significance to this field.

Under the pressure of traffic congestion, the central government and local governments in China have continuously discussed and introduced a series of anti-congestion policies. The Ministry of Transport has stated that efforts should be made to build an urban public transport system and increase investment in bus infrastructure and hub stations to solve the problem of urban congestion [8]. Beijing has also issued the Opinions on Further Promoting the Work of Easing Traffic Congestion in the Capital, which formulates comprehensive measures from the following five aspects: improving urban planning, speeding up road traffic infrastructure construction, prioritizing public transportation development, improving the non-motor vehicle traffic system, and strengthening the management and scientific management of motor vehicles [9]. In response to congestion and with the aim of achieving a speed of no less than 25 km/h on central urban roads within 5 years, Guangzhou has issued 30 ordinances, including raising parking fees, studying the levy of traffic congestion fees, and studying the prohibition of foreign cars into the city [10].

Traffic congestion, as a trending urban problem, has also been applied in SD studies in recent years. For example, Wang et al. studied the SD model of an urban traffic system and its application. Building on the SD method, the authors established a model of the urban traffic system based on system structure analysis and causality feedback analysis [11]. Furthermore, Shuang conducted an empirical study on the evolution mechanism of urban traffic structures based on SD [12]. Finally, Yimei and Yi studied previous SD model simulations of urban traffic congestion, analyzed the formation mechanism of urban traffic congestion, and established an SD model of urban traffic congestion [13].

Unfortunately, the existing SD models of traffic congestion in the aforementioned studies are characterized by macroscopic complexity, numerous internal module elements, and attempts to cover all aspects of the urban economy and society. The main defects are that the setting of the traffic demand generation module is too simple, and some important factors (such as hidden demand) are ignored. In addition, past model construction has often emphasized simulation prediction ability, paid attention to the model parameter check and quantitative simulation result description, and has lacked comprehensive and in-depth elaboration of the model framework. As a result, the simulation process has become a black box due to its extreme complexity and insufficient explanation of the failures of past control policies. More importantly, previous models have ignored the subjective initiative of the government to formulate relevant policies to address specific problems; that is, the effect of traffic congestion on the promotion of anti-congestion policies. Therefore, previous models lacked the inclusiveness of emerging congestion control policies and faced difficulty in evaluating specific policies in a targeted way.

Based on these observations, We propose the following hypothesis:

**Hypothesis 1 :.**

The “Sparse Block Collocation” Policy was probably the most sustainable and fundamental congestion control measure.

Then, this study uses an SD regression to improve the basic goal of the mental model and highlight the analysis function of the SD model to deduce the influence of policy implementation. The SD method is used to analyze current traffic congestion control measures in China and determine which control measures are the most effective and sustainable. By depicting the causal feedback diagram of the congestion mechanism (also known as the system flow diagram, which refers to the organic combination of the basic variables of SD and its symbols, the internal structure of the system, and its feedback mechanism for qualitative analysis), this study answers the following question: Which popular transportation policies are truly effective and sustainable? And the main aim of this work is to provide consult for the government to put forward reasonable and effective measures to control traffic congestion.

## 2. Materials and Methods

### 2.1. SD Method

SD was founded in 1956 by Jay Forrester and is a method used in the quantitative research of complex social and economic systems based on feedback control theory and computer simulation technology [14]. The SD method is realized by establishing a feedback loop (also known as a causal feedback loop) and setting various variables and equations. A feedback loop refers to a closed causal chain sequence composed of different elements or variables in a system. In 1988, McLaughlin published the book Application of Systems Approach to Urban and Regional Planning in which he asserted that “cities can be regarded as dynamic systems that are constantly changing under the influence of many factors, so the planning of cities with dynamic systems must also be dynamic planning with constant changes” [15] (p.105).

Forrester published Urban Dynamics in 1969; however, SD was only introduced in China in 1980, with the first national academic exchange conference on the topic held in 1986. Since then, many institutions have both conducted research on and considered the applications of this concept [16]. For example, in the field of urban transportation, studies have examined private car ownership in China. Li and Yaping studied urban traffic development using the dynamics model of the private car consumption system in Beijing. It was based on system structure and causal feedback analysis; and it discussed the restraining effect of the development of public transportation, especially rapid rail transit, and private cars development; and provided a decision-making basis for the formulation of an urban transportation development policy [17]. The models and methods mentioned above are similar and develop gradually, providing inspiration and reference for this study.

### 2.2. Building the Formation Mechanism Model of Traffic Congestion

**Figure 1:**
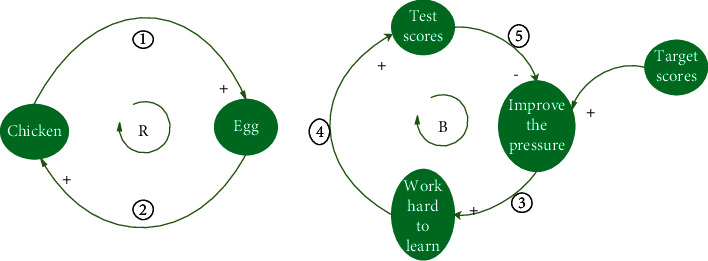
Example of reinforcing loop (a) and balancing loop (b).

The most important aspect of drawing a causal feedback map of congestion mechanisms is to understand that there are two feedback loops in the system: positive and negative. Each feedback loop is composed of more than two variables with a clear causal relationship between the variables, and there are two kinds of positive (+) and negative (-) causal relationships. A positive feedback loop refers to when the number of negative causalities in the feedback loop is even. A negative feedback loop is one in which the number of positive causalities is odd. Positive feedback loops are characterized by self-enhancement and self-amplification [18]. Typical examples are the chicken–egg and egg–chicken (①⟶②⟶①, Figure 1, a). Negative feedback loops are self-controlled and self-weakening. A typical example is an effort made to achieve a certain goal. For example, students can determine their future degree of study efforts according to the gap between their test scores and target scores to ensure that their academic performance will be closer to their target through their constant effort (③⟶④⟶⑤, Figure 1, b). In this study, the comprehensive judgment of traffic congestion is the result of a cycle caused by urban construction and traffic environment deterioration under the guidance of a self-enhancement mechanism.

### 2.3. Method of Analysis

#### 2.3.1. Operational causal chain

**Definition 1 .**

If the simulation equation of the causal chain from the variable X(t) to the dependent variable Y(t) is the function Y(t) = f(X(t)), wherein f is the operation symbol of the function, then the causal chain $X\left(t\right)\overset{f}{⟶}Y\left(t\right)$ that gives the simulation operation symbol is called the operation causal chain.

**Definition 2 .**

If we run the causal chain $X\left(T+k*DT\right)\overset{f}{⟶}Y\left(T+k*DT\right)$, it is called the causal chain of simultaneous operation of simulation step size. If the operation causal chain is $X\left(T+\left(k-1\right)*DT\right)\overset{f}{⟶}Y\left(T+k*DT\right)$, it is called the causal chain of simulation step length heteroscale operation.

*Flow level variable simulation step length multi-layer operation causal chain*. Since the flow bit *L*_*i*_(*t*) has the following function relationship:

(1) $$\begin{array}{c} L_{i}\left(T+k*DT\right)=L_{i}\left(T+\left(k-1\right)*DT\right)+\\ DT\;*R_{i1}\left(T+\left(k-1\right)*DT\right)-DT\;*R_{i2}\left(T+\left(k-1\right)*DT\right). \end{array}$$

Then T is the starting time of simulation, and the left side of the function of the flow bit variable *L*_*i*_(*t*) is the simulation step length of *t*=*T*+*k∗DT*, and the right side is the simulation step time of *t*=*T*+(*k* − 1)*∗DT*, with a difference of one simulation step DT.

The value of *L*_*i*_(*T*+*k∗DT*) on the left side of the flow potential variable is the algebraic sum of the three terms on the right side of *L*_*i*_(*T*+(*k* − 1)*∗DT*), the inflow rate *R*_*i*1_(*T*+(*k* − 1)*∗DT*), and the outflow rate *R*_*i*2_(*T*+(*k* − 1)*∗DT*). There are three corresponding causal chains from right to left:

(2) $$\begin{array}{c} L_{i}\left(T+\left(k-1\right)*DT\right)⟶L_{i}\left(T+k*DT\right),\\ R_{i1}\left(T+\left(k-1\right)*DT\right)⟶L_{i}\left(T+k*DT\right),\\ R_{i2}\left(T+\left(k-1\right)*DT\right)⟶L_{i}\left(T+k*DT\right). \end{array}$$

The three chains from right to left of the flow potential variable are endowed with different operations to form the algebraic sum of the three terms and the functional expression:

① *L*_*i*_(*T*+(*k* − 1)*∗DT*)⟶*L*_*i*_(*T*+*k∗DT*), which is the addition of the three algebraic sums, the “+” operations, and the causal chain with the “+” symbol:

(3) $$\begin{array}{c} L_{i}\left(T+\left(k-1\right)*DT\right)\overset{+}{⟶}L_{i}\left(T+k*DT\right). \end{array}$$

In this study, “+” in the causal chain represents the “plus” operation symbol, but this does not mean that the causal chain polarity is positive. The “-” is in the middle of the causal chain and represents the “minus” sign in the operation. The same applies to the operations below.

② *R*_*i*1_(*T*+(*k* − 1)*∗DT*)⟶*L*_*i*_(*T*+*k∗DT*) is the addition to the algebraic sum of three terms and is also the plus term. However, there is an operation term of *∗DT* again, so there is a causal chain with a “+ “ and “ *∗DT*“ double operation symbol. It is marked as follows:

(4) $$\begin{array}{c} R_{i1}\left(T+\left(k-1\right)*DT\right)\overset{+,*DT}{⟶}L_{i}\left(T+k*DT\right). \end{array}$$

③ *R*_*i*2_(*T*+(*k* − 1)*∗DT*)⟶*L*_*i*_(*T*+*k∗DT*) is the subtraction item in the three-term algebraic sum and is the “-” operation item and also the operation item with *∗DT*, so there is a causal chain with a “-” and “ *∗DT*“ double operation symbol.

(5) $$\begin{array}{c} R_{i2}\left(T+\left(k-1\right)*DT\right)\overset{-,*DT}{⟶}L_{i}\left(T+k*DT\right). \end{array}$$

**Figure 2:**
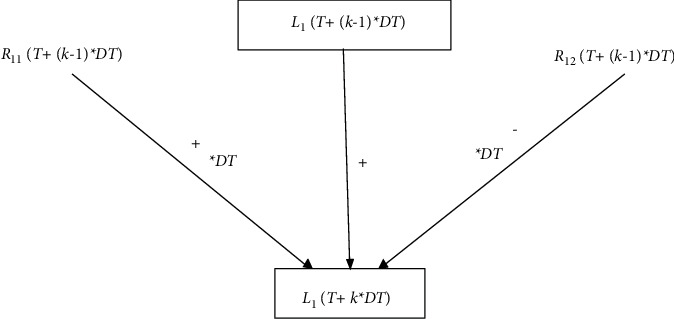
Flow level variable *L*_*i*_(*T*+*k∗DT*) of the different layer operation causal chain diagram.

The above three operation causal chains reveal that the causal chain of the flow potential variable *L*_*i*_(*T*+*k∗DT*) is composed of the following three causal chain diagrams of the different layer operations.  Figure 2 shows that the vertex of the flow potential variable in the calculation causal chain graph is the frame vertex in the hold flow graph.

**Proposition 1 .**

The causal chain of flow potential variables operation in the system dynamics flow diagram is all 3-layer operation causal chain of flow potential variables *L*_*i*_(*T*+*k∗DT*). It is the combination of the causal chain of the flow potential variables in the flow diagram and the simulation equation: *L*_*i*_(*T*+*k∗DT*)=*L*_*i*_(*T*+(*k* − 1)*∗DT*)+*DT*  *∗R*_*i*1_(*T*+(*k* − 1)*∗DT*) − *DT*  *∗R*_*i*2_(*T*+(*k* − 1)*∗DT*).

Intuitively revealing the causal chain of different level operation, the value of the flow level variable *L*_*i*_(*T*+*k∗DT*) is its last moment *L*_*i*_(*T*+(*k* − 1)*∗DT*) value, plus the inflow rate times the step size*DT*  *∗R*_*i*1_(*T*+(*k* − 1)*∗DT*) value, and the minus outflow rate times the step size *DT*  *∗R*_*i*2_(*T*+(*k* − 1)*∗DT*) value. Combining the causal chain with the simulation equation, the internal relationship between the causal chain and the simulation equation can be established, and the intuitiveness of the model can be enhanced.

Flow rate variable simulation step length time simultaneous operation causal chain. After the simulation calculation of flow level variable of the system dynamics model, the simulation calculation of flow rate variable is carried out, and the simulation calculation of flow rate variable is the same as the simulation step length calculation. For example, the quotient equation of outflow rate *R*_*i*2_(*t*): *R*_*i*2_(*t*)=*L*_*i*_(*t*)/*D*_1_. The independent variables on both sides of the equation are t, and in the flow diagram, there exists a causal chain from the flow potential variable to the outflow rate:

(6) $$\begin{array}{c} L_{i}\left(t\right)⟶R_{i2}\left(t\right). \end{array}$$

The operator of this quotient equation is 1/*D*_1_, at the *t*=*T*+*k∗DT* simulation step length, the causal chain of its parallel operation is:

(7) $$\begin{array}{c} L_{i}\left(T+k*DT\right)\overset{1/D_{1}}{⟶}R_{i2}\left(T+k*DT\right). \end{array}$$

The flow rate causal chain is the combination of the flow level variable to the flow rate causal chain and its simulation equation.

According to the definition, in the flow diagram of system dynamics, flow rate to flow rate, flow rate to auxiliary variable, auxiliary variable to flow rate, and auxiliary variable to auxiliary variable are all causal chains of the same layer operation.

**Proposition 2 .**

In the dynamic flow diagram of the system, the causal chain of the most operation of flow level change is the causal chain of the simulation step length and time different layer operation, and the causal chain of the other variable operation is the causal chain of the simulation step length and time the same layer operation.

**Proposition 3 .**

System dynamics any simulation step length time *t*=*T*+*k∗DT*. First of all, according to the causal chain of each flow potential variable with 3 different levels of operation, the *T*+(*k* − 1)*∗DT* correlation multiplier of the previous simulation step is used to calculate the causal chain of each flow potential variable, and the values of all flow potential variables *t*=*T*+*k∗D* time are calculated. Then, do the same level operation for the other variables, the causal chain calculation, and calculate the values of *t*=*T*+*k∗D* time for all the other variables.

The above three propositions are the default requirements of the system dynamics simulation program.

#### 2.3.2. Operation process diagram

*Definition of operation process diagram*. Let *G*=(*V*(*t*), *X*(*t*)) be an ordered binary, *V*(*t*) is the vertex set, *V*(*t*)={*V*_1_(*t*), *V*_2_(*t*),…, *V*_*n*−1_(*t*), *V*_*n*_(*t*)}; *X*(*t*) is the directed arc set, an arc is an ordered pair of (*V*_*i*_(*t*), *V*_*j*_(*t*)) composed of different elements of *V*_*i*_(*t*) · *V*_*j*_(*t*) of the vertex set *V*(*t*). Then *G*=(*V*(*t*), *X*(*t*)) is called a directed graph.

**Definition 3 .**

directed graph *G*=(*V*(*t*), *X*(*t*)), t is the simulation step length time *T*+*K∗DT*, *V*(*T*+*K∗DT*) is the vertex set of data process, arc set *X*(*T*+*K∗DT*) is the set of operation causal chain of assignment operation symbol, an ordered binary set of vertex sets of data procedures and causal chain sets of operations is called an operation procedure graph, recorded as *G*=(*V*(*T*+*K∗DT*), *X*(*T*+*K∗DT*)).

**Definition 4 .**

The computation process diagram of the simulation model is an ordered binary of the vertex set of the data process and the causal chain set of the computation.

*The establishment steps of operation process diagram*

**Step 1 .**

Based on the simulation table or some known conditions, the causal chain of flow graph variable is combined with the simulation equation, and the operational causal chain graph without calculating the vertex value is established layer by layer.

**Step 2 .**

The variable data process of calculating the uncalculated vertex values of each layer of the causal chain graph, showing the calculation process of variable vertex values.

## 3. Results and Discussion

### 3.1. The Logic of Traditional “Road Construction and Congestion Control” Policies

From the perspective of SD, this section describes two disadvantages of traditional road construction and congestion control strategy: the release of short-term hidden demand and the generation of medium - and long-term demand. To some extent, this opens the analysis and evaluation of several congestion control measures in the following discussion.

The direct cause of traffic congestion is an imbalance between the demand for car travel and the supply of road facilities. In recent decades, China's urbanization process has accelerated. An increasing number of people have entered cities, and living standards have been continuously improved. Private cars have gradually transformed from high-end luxury goods to mass-consumption goods. Under the combined force of an increase in the population base and a rise in the private car ownership rate, the demand for car travel has increased, thereby rendering transportation infrastructure in many cities insufficient to support this shift, resulting in added traffic congestion [19].

Regarding public complaints, the government's pressure on congestion control has increased, and road construction has long been the dominant solution. Road widening, viaduct construction, expressway construction around the city, and other unimpeded projects are common in various cities in China. In addition, driven by the desire for image projects and urban expansion, Chinese city governments invest billions in road planning, construction, and management annually. This infrastructure is given to the car group free of charge, along with many modern traffic managers and management methods. Furthermore, 70% to 80% of traffic researchers' efforts serve car users [20].

**Figure 3:**
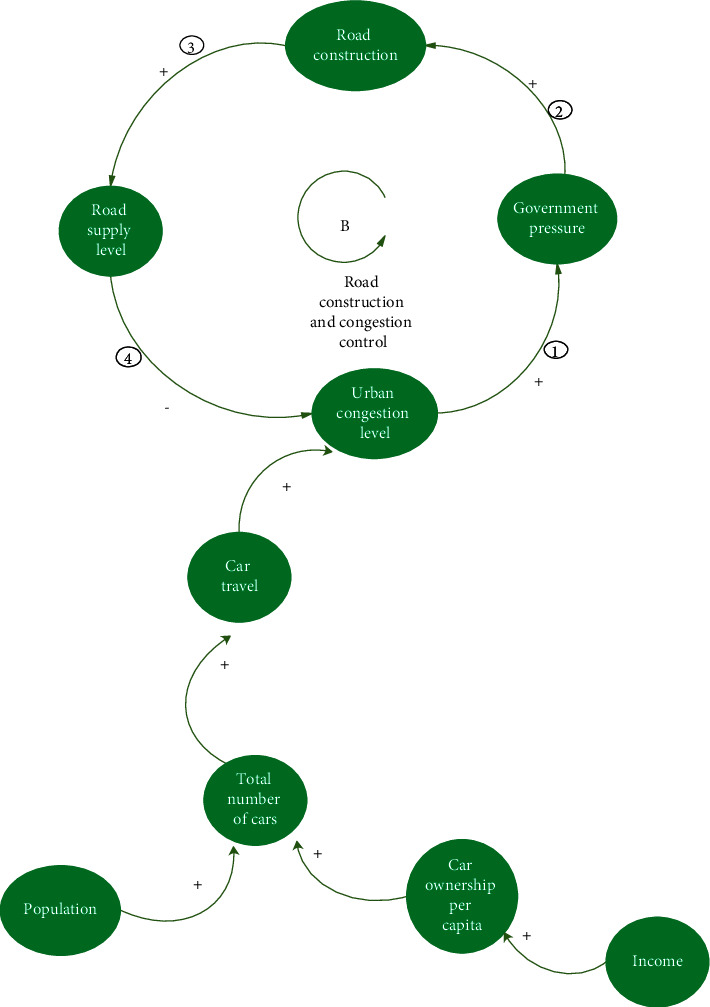
Conventional logic of building more roads to relieve congestion.

From the perspective of SD, the causality of ①⟶②⟶③⟶④ groups form a closed negative feedback loop, thereby indicating that road construction can alleviate local traffic congestion in the short term (Figure 3). If traffic was simply a self-debilitating mechanism as many government officials and technologists imagine it to be, then the problem would be solved. However, this is not the case because there is a series of self-reinforcing mechanisms that play a more dominant role.

#### 3.1.1. Post-Road Effect 1: The Release of Short-Term Hidden Demand

**Figure 4:**
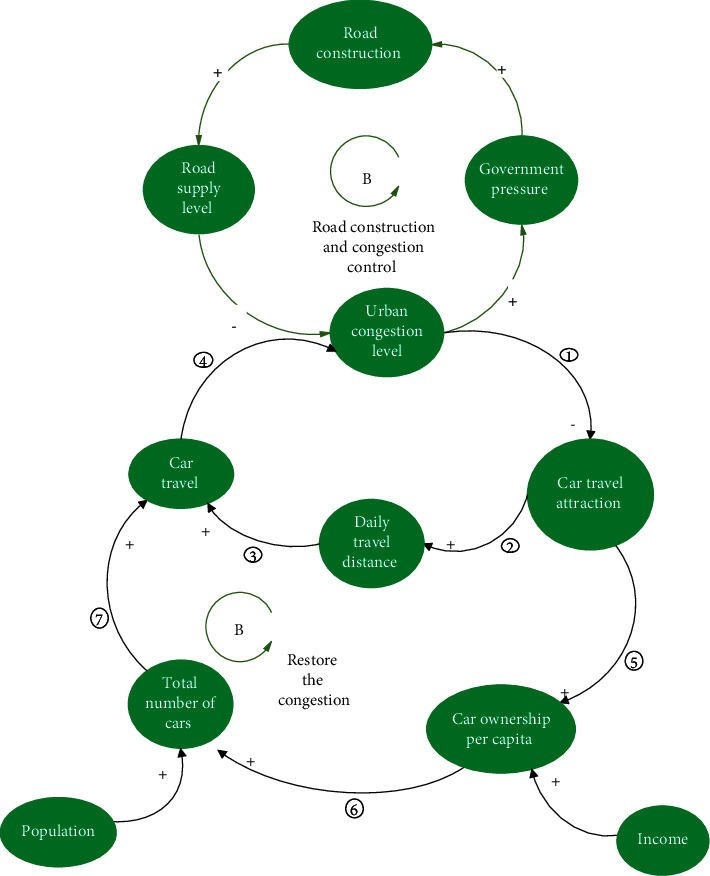
Short-term release of latent demand after road building.

When the government was occupied by the positivity of road construction and ribbon cutting, urban congestion was alleviated immediately. However, in the short term, the appeal of car travel was rapidly improved, which directly encouraged people to drive both more frequently and for longer distances, thereby leading to the increase of car travel and the subsequent congestion of new roads (negative feedback loop ①⟶②⟶③⟶④, Figure 4). Conversely, the appeal of car travel became the most important factor in encouraging citizens to buy private cars, other than an increase in income, which also led to an increase in car travel volume and the deterioration of road congestion (negative feedback loop ①⟶⑤⟶⑥⟶⑦⟶④, Figure 4). Figure 4 shows that there are two negative feedback loops in the process of eliminating the congestion control effect caused by road construction, which indicates that the suppressed hidden demands of the original congestion period are released and transformed into explicit demands, thus aggravating the road burden.

However, the four-stage traffic model of “trip generation - trip distribution - mode division - road network allocation,” widely adopted by transportation professionals, cannot accurately simulate the hidden demands, which leads to the conclusion that more roads can solve congestion. Although traffic modelers assume future increases in travel demand in traffic forecasting scenarios, these demands are often considered the product of economic growth and car ownership. In this model, expanding the supply of road infrastructure does not affect the forecast of future transport demand growth [21]. In other words, the importance of latent demand has been ignored.

#### 3.1.2. Post-Road Effect 2: Generation of Medium- and Long-Term Derivative Demand

**Figure 5:**
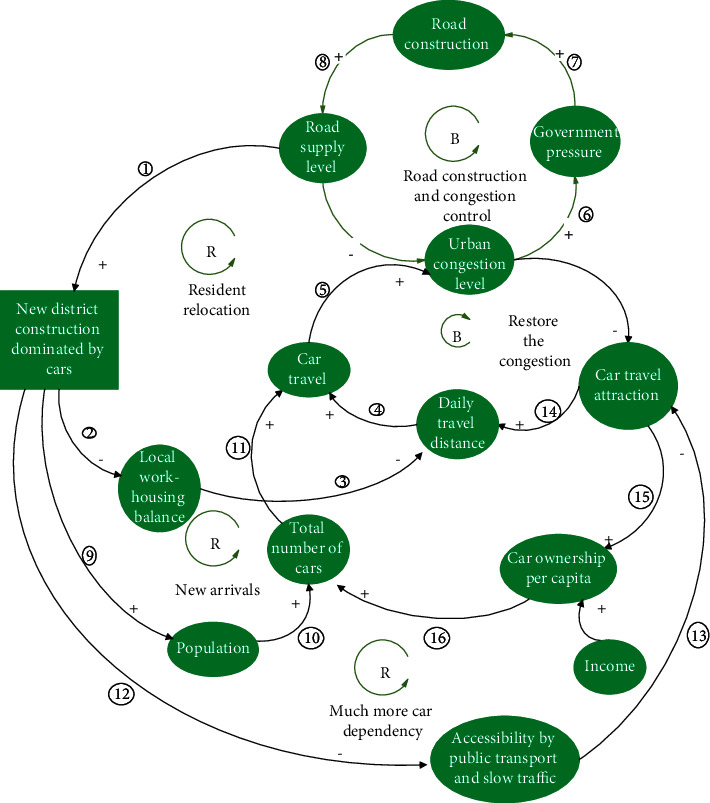
Medium- and long-term generation of induced demand after road building.

In the medium- and long-term perspectives, road construction will also lead to a cycle of “congestion, road construction, congestion, and road construction” (Figure 5) because the government builds roads on the outer edge of the city to enclose land. This construction usually adopts a car-led development model, thereby resulting in a large amount of induced demand. Three positive feedback loops account for the rapid deterioration of urban traffic congestion in China, as follows.

First, the imbalance between jobs and housing in new areas led to the emergence of several sleeping cities around central cities (such as Huilongguan and Tiantongyuan in Beijing). The migration of residents has greatly increased the daily commuting distance, which causes congestion. However, the government continues to build roads to control congestion, which causes a vicious cycle (positive feedback loop ①⟶②⟶③⟶④⟶⑤⟶⑥⟶⑦⟶⑧⟶①, Figure 5).

Second, large new urban areas accommodate more urban populations, thereby objectively increasing the total population base of urban travel and the congestion of the traffic system. Highstreets and large road networks lead to low permeability of bus systems, and meeting client satisfaction regarding speed and efficiency is a common problem. Regarding the slow traffic environment, the dredging and construction of the main roads block the microcirculation of the slow traffic network, resulting in the need for a long-distance detour to avoid crossing the road. However, intersections of wider roads form larger intersections, which causes excessively long waiting times at red lights, traffic disorder at intersections, and the need to climb the overpass under the underground passage; all of which causes inconvenience. Additionally, the car parking management in the new districts is loose, and the phenomenon of bicycle lanes and sidewalks being occupied by parked cars is prominent. Furthermore, because of the lack of bicycle parking facilities, parking is difficult, and it is easy to lose bicycles at destinations due to poor parking management. Subsequently, bicycles have become the second transportation choice, even for short- and medium-distance travel, thereby augmenting the reliance on car travel (positive feedback loop ①⟶⑫⟶⑬⟶⑭⟶④⟶⑤⟶⑥⟶⑦⟶⑧⟶①, Figure 5).

Similarly, the third loop involves the deterioration of public transportation and slow-moving environments and has also led to a surge in the car ownership rate in new districts. Many people regard staying in large districts and buying private cars as binding lifestyle transition nodes (positive feedback loop ①⟶⑫⟶⑬⟶⑮⟶⑯⟶⑪⟶⑤⟶⑥⟶⑦⟶⑧⟶①, Figure 5).

The three positive feedback loops outlined above are the main reasons for the rapid deterioration of urban traffic congestion in China. Taking Beijing as an example, in the last 10 years, the functions of Beijing have been concentrated in the central urban area, thereby resulting in a large increase in the proportion of long-distance travel that is difficult to complete on bicycles. According to a survey by the Beijing Transportation Development Research Center, the proportion of bicycle trips in Beijing dropped from 30.3% in 2005 to 17.9% in the first half of 2019 [22]. Among the car trips, the proportion of short-distance trips within 5 km was more than 40% [23]. The reason for driving is because the environment for walking and cycling is deteriorating, which is closely related to the new urban form that is dominated by large blocks and wide roads.

The above analysis shows the mechanism of the release of implicit demand for car travel and the generation of the derivative demand in the post-road construction period. It can be predicted that when per capita income reaches a point where it is no longer a major barrier to household car consumption (for a significant portion of households), by allowing the ability to choose to travel by motor vehicle, traffic congestion will follow a barrel effect with slow bus accessibility as a shortcoming.

### 3.2. Analysis of Several Congestion Control Policies

According to the framework in the above section, the congestion control policies can be divided into five categories: similar road construction, rigid intervention, supply adjustment, demand guidance, and sparse block collocation. Using the framework established in the previous section, we next analyze the congestion control policies.

#### 3.2.1. Similar Road Construction Policies

**Figure 6:**
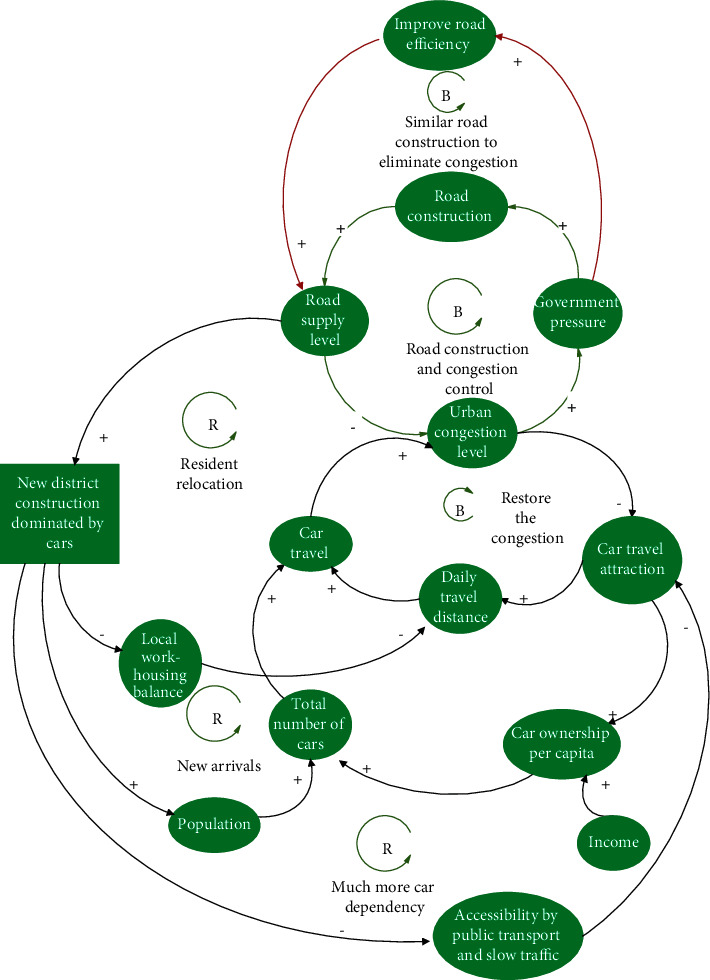
Consequences of adopting similar road construction policies.

Such policies include increasing the supply of parking spaces, improving the service level of intersections (building flyovers and pedestrian overpasses), enhancing the efficiency of road networks (using intelligent transportation systems and creating green wave corridors for vehicles), and so on [24]. The goal of these policies is to increase the space supply of motor vehicles or expand the capacity of road traffic by summarizing them as “similar road construction and congestion control” policies. Similar to road construction, closing the negative feedback loop indicates that the road supply level can indeed increase in the short term. However, it will soon be consumed by the released hidden demand and the newly-derived demand, thereby exacerbating traffic congestion further (Figure 6).

#### 3.2.2. Rigid Intervention Policies

Rigid intervention policy measures include population control, license plate lottery, and car license plate restrictions, which are characterized by direct and forcible interventions in the growth of key elements within the traffic system (such as population and cars or car travel volume). Although these measures will have an immediate effect in the short term, they are often conducted at the expense of higher personal and social costs and are suspected of tending toward groups with vested interests. For example, after the restrictions on these key elements were implemented, the need to shift to the public transport system made the already crowded situation worse [25]. Meanwhile, such policies only temporarily transform part of the explicit demand into implicit demand, which is likely to be re-released when the policy is relaxed, thereby resulting in the rapid recovery of the system and the aggravation of congestion.

Many drivers and traffic police groups attribute traffic congestion to the low civilization quality of pedestrians and cyclists (such as running red lights, jaywalking, etc.), and believe that traffic civilization construction can be strengthened to alleviate traffic congestion [26]. However, without discussing the effectiveness and lag of the construction of traffic civilization, this can be attributed to the following reasoning. If from tomorrow, citizens begin to strictly observe the traffic order (such as not running red lights), then the traffic order may be greatly improved in a short time, thereby significantly improving the driving efficiency of motor vehicles. However, this improvement can only be made possible by inconveniencing pedestrian or cycle travel (such as the introduction of a long wait at traffic signals), which, in the long term, will encourage people to drive, once they are able to buy a car, thus adding to the congestion[27].

#### 3.2.3. Supply Adjustment Policies

**Figure 7:**
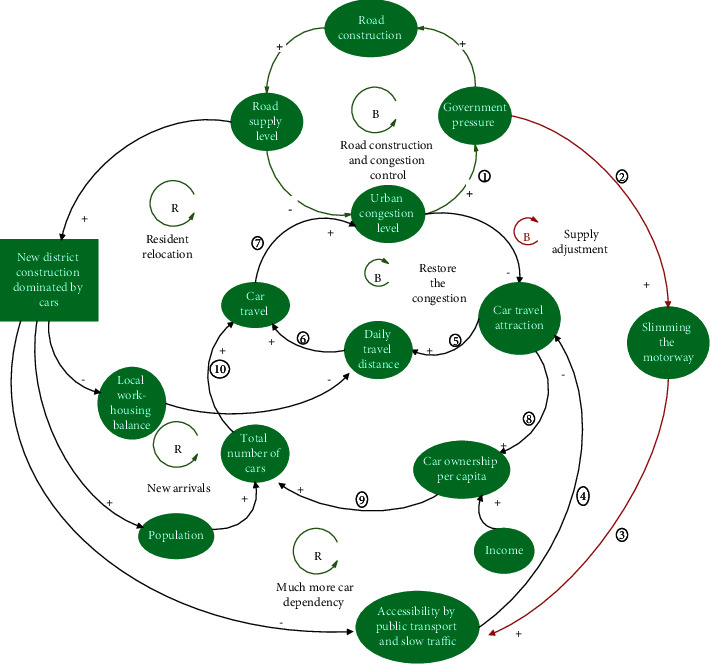
Consequences of adopting adjusting supply policies.

In contrast to the “rigid intervention type” policies, supply adjustment policies start from the “supply side,” prioritize slow or public transportation and attempt to introduce a new negative feedback loop to achieve a self-reduction mechanism of congestion (Figure 7). By reducing unnecessary vehicle space (such as avoiding excessively wide vehicle lanes, reducing the turning radius of intersections, and adding safety islands), the crossing distance for slow pedestrians can be shortened, the speed of vehicles can be reduced, and the safety and convenience of walking and cycling can be improved. This result will indirectly improve the accessibility of the public transportation system and reduce the dependence on cars, cars' travel distances, and the rate of private car ownership, thereby reducing the number of car trips and alleviating traffic congestion (negative feedback loop ①⟶②⟶③⟶④⟶⑤⟶⑥⟶⑦, Figure 7; negative feedback loop ①⟶②⟶③⟶④⟶⑧⟶⑨⟶⑩⟶⑦, Figure 7).

**Figure 8:**
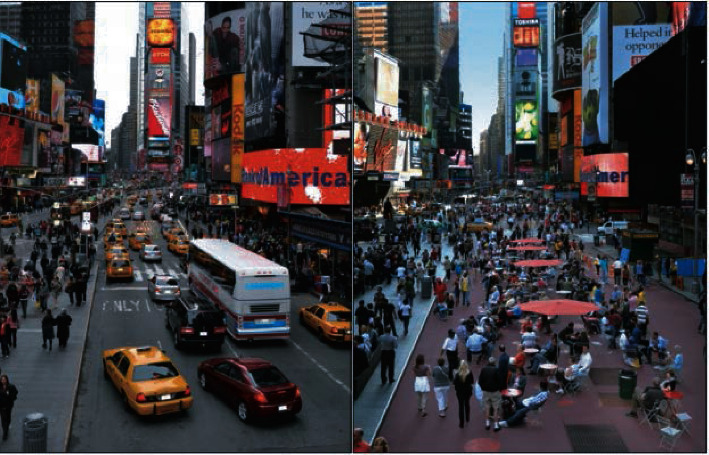
Before/after comparison of the Broadway renovation project in New York.

The slimming of roads may seem to reduce the amount of space available for vehicles, but it does not. So, by optimizing the organization of roads and effectively reducing the interference of mixed traffic, it is possible to achieve a win-win situation for slow traffic and motor vehicles. In recent years, some foreign cities (such as New York, Copenhagen, Seoul, etc.) have boldly transformed urban motorways into slow lanes or bus lanes. As a result, they have not only improved the vitality of the city and the quality of public spaces but have also eased traffic congestion, which has been welcomed by citizens. For example, the renovation project of Broadway Avenue in New York transformed a congested road into a special strip for pedestrians to walk and rest (Figure 8). According to a recent evaluation study, the speed of vehicles in the area increased by nearly 17% after the project was implemented [28].

**Figure 9:**
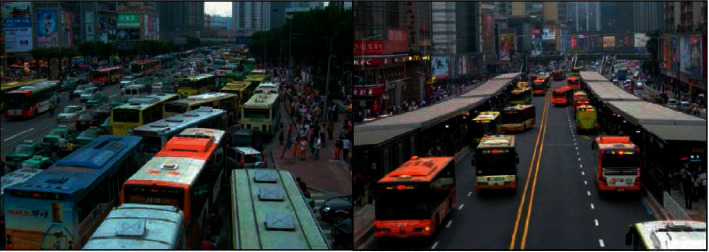
Before/after comparison of the Zhongshan Avenue renovation in Guangzhou.

Another example is the Bus Rapid Transit (BRT) test line in Guangzhou, which was built in the most congested area where buses and social vehicles had serious interaction before its reconstruction. By turning the motor lane in the middle of the road into a bus lane, the running speeds of both public and social vehicles were improved (Figure 9). After the BRT corridor was put into operation in February 2010, the average speed of social vehicles along Zhongshan Avenue increased by 28% compared to before [29].

#### 3.2.4. Demand-Oriented Policies

**Figure 10:**
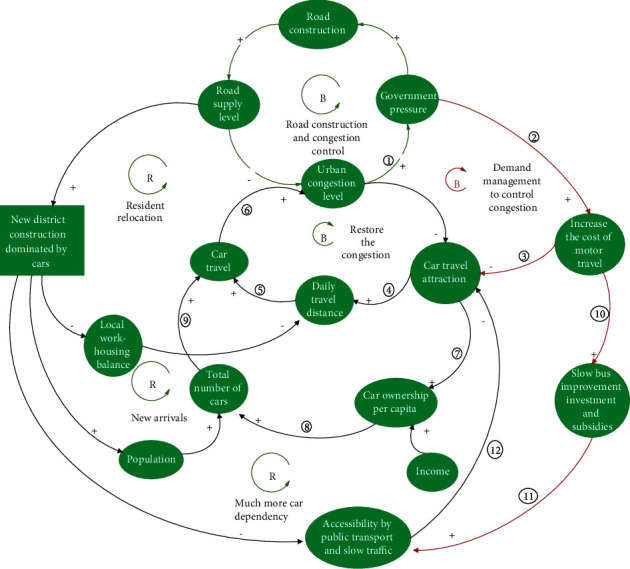
Consequences of adopting demand-oriented policies.

From the perspective of demand management, the policies of economic leverage to increase cars' travel costs have increased parking fees, levied congestion charges, and strengthened the restrictions on unregulated parking. These measures can effectively restrain the uncontrolled use of cars during rush hour (negative feedback loop ①⟶②⟶③⟶④⟶⑤⟶⑥, Figure 10) and can simultaneously slow down the popularity of cars, reduce the number of car trips, and alleviate the degree of congestion (negative feedback loop ①⟶②⟶③⟶⑦⟶⑧⟶⑨⟶⑥, Figure 10).

Another benefit of increasing parking fees and congestion pricing is the amount of economic revenue that the city government can generate, which can then be used to subsidize the construction or improvement of the efficiency of the public transport system [30]. It can also improve the slow travel environment, increase the attractiveness of short- and medium-distance slow travel, and solve the last mile problem for long-distance bus travel. All these changes will reduce citizens' reliance on cars for travel, the distance traveled by cars, the rate of private car ownership, and car trips, thereby alleviating congestion (negative feedback loop ①⟶②⟶⑩⟶ 11 ⟶12 ⟶④⟶⑤⟶⑥, Figure 10; negative feedback loop ①⟶②⟶⑩⟶ 11 ⟶12 ⟶⑦⟶⑧⟶⑨⟶⑥, Figure 10). Similar measures have been adopted in London since February 2003, where part of the revenue from traffic congestion fees have been used to improve public transport in the region. As a result, not only did 20% fewer cars enter the city center every day, but buses were also 25% faster than before, thus reducing the delay time of motor vehicles in the congested charging areas during peak hours by 30% (Figure 10) [31].

#### 3.2.5. Sparse Block Collocation Policies

With the aim of increasing the construction of public transport systems and adopting the transit-orientated development (TOD) model in the construction of new areas around bus stations, the policies are to: (1) design walkable streets and pedestrian-scale blocks to enhance pedestrian traffic; (2) incorporate pedestrian safety and convenience requirements into architectural design; (3) reduce the demand for motor vehicles by creating bicycle-friendly road networks; (4) increase the use of public transport by building public transport-oriented streets and communities; (5) advocate mixed land-use patterns to disperse public travel destinations; and (6) establish public green spaces and services within walking distance of each other [32].

**Figure 11:**
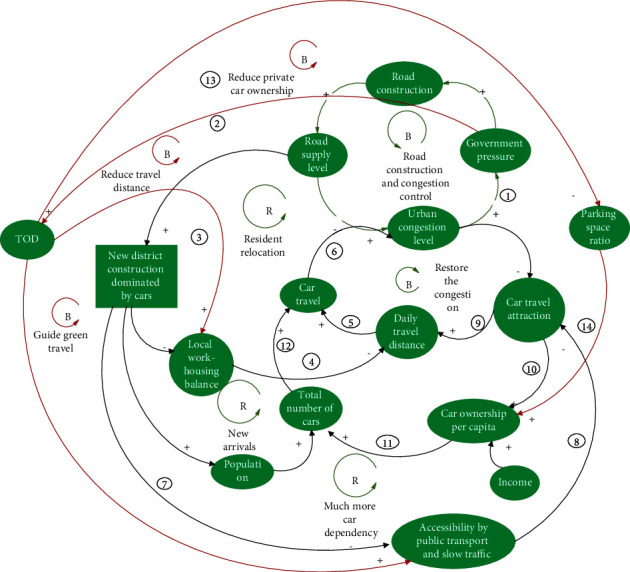
Consequences of adopting TOD policies on the traffic demand and supply simultaneously.

The impact of such policies can be explained by the following series of negative feedback loops (Figure 11).

Mixed land use enables more balanced employment and housing, shortens commuting distances, and reduces traffic demand. Simultaneously, it is possible to use bicycles and walk for short and medium distances, which increases the proportion of green travel and reduces car travel (negative feedback loop ①⟶②⟶③⟶④⟶⑤⟶⑥, Figure 11).

Increasing the construction of public transportation facilities and attractions of public transportation can reduce the dependence on cars. Owing to the distribution of attraction points along the bus corridor, pendulum traffic flow can be avoided. The reduction of the one-way distance per person can improve the bus carrying turnover rate and avoid one-way traffic congestion during peak hours. The development of small blocks can reduce bottlenecks along major and secondary roads and large intersections, thereby enhancing the permeability of public transportation services and solving the problem of the last mile by improving the slow traffic environment. The impact of these aspects can reduce the attractiveness of car travel by lowering the private car ownership rate, car use intensity, and a number of car trips, thereby alleviating traffic congestion (negative feedback loop ①⟶②⟶⑦⟶⑧⟶⑨⟶⑤⟶⑥, Figure 11; negative feedback loop ①⟶②⟶⑦⟶⑧⟶⑩⟶ 11 ⟶12 ⟶⑥, Figure 11).

If the number of parking spaces is reduced and the private car ownership rate of residents in TOD areas can be effectively controlled to a low level, the number of car trips can be reduced and congestion alleviated (negative feedback loop ①⟶②⟶ 13 ⟶14 ⟶11 ⟶12 ⟶⑥, Figure 11).

Compared with previous types of congestion control policies, public transit-guided development and sparse block collocation policies can establish multiple and effective congestion mitigation mechanisms from “open sources” (attracting car travel to other modes of transportation) to “throttling” (reducing basic travel volume), thereby creating more comprehensive and further-reaching traffic control.

## 4. Conclusions

This study builds an urban congestion mechanism model based on SD theory. The analysis shows that although traditional road construction and control congestion methods can alleviate congestion in a short period of time, the huge hidden demand and the development of car-oriented new districts caused by road construction will be prevalent in the future. Under the dual influence of the derived demand, traffic congestion will quickly recover and intensify. Unfortunately, traffic congestion cannot be solved by traffic engineers alone, as traditional traffic model predictions cannot simulate these interactions.

Faced with the pressure to control congestion, the government has adopted five policy types: similar road construction, rigid intervention, supply adjustment, demand guidance, and sparse block collocation. This study introduces the SD model to analyze these policies and shows that similar road construction policies continue to misunderstand traditional road building to prevent traffic congestion. Although rigid intervention policies have immediate effects in the short term, they will be at the expense of personal convenience and social cost, and the suppressed hidden demand will eventually be released, making it unsustainable overall. The supply-adjusted policies and demand guidance policies are relatively more effective, not only for short-term results but also for long-term sustainability. Meanwhile, the sparse block collocation policies are the most sustainable and fundamental congestion control measure, with public transport-oriented land use and development as its core. It forms multiple negative feedback loops to relieve traffic congestion from both the supply and demand aspects. Although the land development policy is often regarded as a long-term policy, it is both urgent and necessary to take effective measures to curb its use, and instead, implement the concept of public transport-oriented development in urban planning policy formulation in view of the rapid development of new areas dominated by cars in China at present.

The fundamental idea of congestion control should be people-oriented, not car-oriented. In the debate between mobility and accessibility, the government has always pursued the mobility of the road, which practices the neglect of essentials. Some officials have argued that the fundamental goal of tackling urban traffic congestion is to guarantee the basic right of residents to travel conveniently, including the freedom to travel by car [33]. In many cases, urban decision-makers often verbally advertise “slow travel first and people-oriented” congestion control measures; however, the ultimate goal is to increase the speed of motor vehicles. This fact is based on the premise of “not harming the interests of car travel” as opposed to the premise of “slow driving and people-oriented” measures, which would be accused of increasing blockages [34]. This logic ignores the harsh reality that given the number of people and high densities of China's cities, the rapid growth of China's economy, and the shortage of oil resources, there is probably no solution that will ultimately satisfy the right of every Chinese residents to drive.

It should be recognized that a small number of people in cities travel purely for the enjoyment of transportation and that most people travel to attain social, recreational, educational, employment-related, and other opportunities and benefits from destinations [35]. Therefore, the government's focus should not be on keeping roads clear (increasing mobility), but on improving accessibility and providing the easiest and cheapest way for citizens to travel. Scientific urban planning and appropriate public policies can achieve twice the result with half the effort. This study found that the sparse block collocation policy is the most sustainable strategy, but did not analyze the strategy's implementation details. Future research should start with the concrete implementation details of the sparse block collocation policy.

## Data Availability

The data used to support the findings of this study are available from website of National Bureau of Statistics.

## References

[1] Beijing Times (2011). Survey says over 80% of car owners suffer from “traffic jam dysphoria. http://www.ckxxbao.com/baozhi/jinghuashibao/(accessedon23April2011.

[2] Minghua G., Zhenghe Z. (2009). The solution to the problem of traffic congestion in big cities. *City Prob*.

[3] Yuanqing W., Linyan S., Chuanjiao S. (2007). The system cause and solution of traffic problem in big cities of our country. *City Prob*.

[4] Xianglin H., Changyuan J., Hongxia G. (2007). Coupled mapping following model and traffic congestion control based on intelligent transportation system. *Journal of Physics*.

[5] Shenzhen (2011). NPC deputies say heavy traffic is not the root cause of congestion. http://epaper.southcn.com/nfdaily/html/2021-06/18/node_2.htm.

[6] Feng L., Runlong H., Jinhong D. (2011). How do megacities regulate population size?. *Population Studies*.

[7] Xianteng L., Qing S., Li Z. (2009). Contradictions between supply and demand of traffic in big cities and countermeasures for their development -- Taking Nanjing as an example. *City and Planning*.

[8] To Solve Congestion (2010). We must vigorously build urban public transport system. http://paper.people.com.cn/rmrb/html/2021-06/18/nbs.D110000renmrb_01.htm.

[9] Beijing Municipal People’s Government (2010). *Opinions of Beijing Municipality on Further Promoting the Scientific Development of Transportation in the Capital and Strengthening the Work of Easing Traffic Congestion (Jing Zhengfa [2010] No. 42)*.

[10] Guangzhou Municipal People’s Government (2011). *Plan for Alleviating Traffic Congestion in Guangzhou Central Urban Area after Asian Games (Draft for Soliciting Opinions)*.

[11] Jifeng W., Huapu L., Hu P. (2008). SD model of urban traffic system and its application. *Transp. Syst. Eng. Inf.*.

[12] Shuang L. (2009). *An Empirical Study on the Evolution Mechanism of Urban Traffic Structure Based on System Dynamics*.

[13] Yimei Z., Yi Z. (2008). Simulation study on system dynamics model of urban traffic congestion. *Transp. Comput.*.

[14] Qifan W. (1998). *System Dynamics*.

[15] Fengwu W. (1998). *Trans. JB McLaughlin. The Application Of Systematic Method In Urban And Regional Planning*.

[16] Teng S. (2009). *System Dynamics Analysis of Planning Policy*.

[17] Liu L., Zhou Y. (2008). Study on urban traffic development based on the dynamic model of private car consumption system in Beijing. *Urban Dev. Res.*.

[18] Cong Z. A., Fl B., Xl A., Yuchuan D (2021). Macroscopic modeling and dynamic control of on-street cruising-for-parking of autonomous vehicles in a multi-region urban road network. *Transportation Research Part C: Emerging Technologies*.

[19] Kamel Boulos M. N., Lu Z., Guerrero P., Jennett C., Steed A. (2017). From urban planning and emergency training to Pokémon Go: applications of virtual reality GIS (VRGIS) and augmented reality GIS (ARGIS) in personal, public and environmental health. *International Journal of Health Geographics*.

[20] Xiaojiang L. (2011). Some thoughts on current urban traffic policy. *Urb. Traff.*.

[21] Sterman J. D. (2000). *Business Dynamics: Systems Thinking and Modeling for a Complex World*.

[22] Guo J., Li X. (2019). *Beijing Transportation Development Annual Report 2019*.

[23] Yong Q., Mingzheng S. (2011). There are several trends in the development of traffic in big cities in China. *Urb. Traff.*.

[24] Tang L., Wang P., Wang C. (2018). Simulation of road transportation energy-saving and emission reduction path based on system dynamics. *Systems Engineering*.

[25] Lin P., Weng J., Hu S., Jing Y., Yin B. (2020). Study on diurnal similarity of individual activity chain of public transport passengers. *Transp. Systems Eng. Inf.*.

[26] You Z. (2004). I analyze the basic causes of urban traffic jam -- from the perspective of management, taking Beijing as an example. *Economics and Management*.

[27] Liangbing F, Zhihan L, Gengchen G, Houbing S (2016). Pheromone based alternative route planning. *Digital communication and networking*.

[28] The New York City Department of Transportation (2010). *Green Light for Midtown Evaluation*.

[29] Weixiong X. (2011). Response measures of tidal passenger flow of bus rapid transit in Guangzhou. *Urb. Traff.*.

[30] Jiangping Z. (2010). Traffic congestion pricing -- the latest international research progress and cases. *City and Planning*.

[31] Victoria Transport Policy Institute (2006).

[32] Fengyue Z., Zhenyu S., Zhigao W. (2011). *Low Carbon Transportation: Chenggong New District, Kunming, China Low-Carbon Eco-City Development Report 2011*.

[33] Guoqing C. (2011). Causes and countermeasures of urban traffic congestion in Beijing. *New Horiz*.

[34] Zhu J., Luo L., Wang D., Ding D. (2017). *Measurement of Residents’ Acceptance of Urban Slow Traffic System: A Case Study of the Pearl River Delta*.

[35] Barton K. (2002). *Transportation Economics*.

